# Changes in species richness and composition of boreal waterbird communities: a comparison between two time periods 25 years apart

**DOI:** 10.1038/s41598-018-38167-1

**Published:** 2019-02-11

**Authors:** Hannu Pöysä, Sari Holopainen, Johan Elmberg, Gunnar Gunnarsson, Petri Nummi, Kjell Sjöberg

**Affiliations:** 1 Natural Resources, Natural Resources Institute Finland (Luke), Joensuu, Finland; 2 Department of Forest Sciences, University of Helsinki, Helsinki, Finland; 3 Department of Environmental Science and Bioscience, Kristianstad University, Kristianstad, Sweden; 4 Department of Wildlife, Fish, and Environmental Studies, Swedish University of Agricultural Sciences, Umeå, Sweden

## Abstract

Global measures of biodiversity indicate consistent decline, but trends reported for local communities are more varied. Therefore, we need better understanding of mechanisms that drive changes in diversity of local communities and of differences in temporal trends between components of local diversity, such as species richness and species turnover rate. Freshwater ecosystems are vulnerable to multiple stressors, and severe impacts on their biodiversity have been documented. We studied species richness and composition of local boreal waterbird communities in 1990/1991 and 2016 at 58 lakes distributed over six regions in Finland and Sweden. The study lakes represented not only local trophic gradients but also a latitudinal gradient in the boreal biome. While species richness tended to be lower in 2016 than in 1990/1991, species turnover was relatively high. Within foraging guilds, local species richness of diving ducks and surface feeding waterbirds decreased, whereas that of large herbivores increased. The number of species gained in local communities was higher in lakes with rich vegetation than in lakes with sparse vegetation. Conservation of boreal freshwater ecosystems would benefit from recognizing that large-scale environmental changes can affect local diversity via processes operating at finer scales.

## Introduction

Recent negative biodiversity trends have been documented for several taxa and for many terrestrial and aquatic ecosystems^1–3^. While global measures of biodiversity indicate consistent decline, and similar trends have been documented for local communities^4,5^, not all recent analyses indicate systematic biodiversity loss at this level^6,7^. Instead, local communities often show variable responses, and those responses may be due to species’ differential reactions to climate change, habitat loss or degradation, compensatory dynamics, or range expansion of alien species^7–10^. Because large-scale changes in biodiversity should reflect the sum of processes operating at the level of local communities, the discrepancy between global and local biodiversity trends is puzzling. Some of the incongruity among studies focusing on local-scale diversity trends may be due to differences in the disturbance history of local communities^4,11,12^. At any rate, recent contradictory findings underscore the need for a better understanding of mechanisms that drive changes in diversity of local communities and for identifying differences in temporal trends among components of local diversity, such as species richness, species turnover rate, and functional diversity^8,13–15^.

Ever since MacArthur and Wilson^16^ developed the influential theory of island biogeography, which is based on the observation that immigration and local extinction affect species richness and composition of local communities, ecologists have acknowledged that diversity of such communities is not static but changes over time^17^. MacArthur and Wilson’s theory emphasized random extinction of small populations as part of natural change in community composition. In addition to this natural process, biological diversity is changing due to anthropogenic stressors, notably habitat loss and degradation, and climate change^5,18–20^. For example, as a result of logging of old-growth forests, animal species richness often decreases and species’ relative abundances change due to decreased structural complexity^21^. On the other hand, poleward shift and expansion of species ranges in response to climate change^22^ may cause species turnover or even species increase in local communities. For example, among birds in northern Europe, both range contractions of cold-dwelling species and expansions of warm-dwelling species have occurred recently^23^. Similarly, the poleward shift in the mean weighted latitude of density of birds breeding in Finland is faster in northern than in southern species^24^.

Eutrophication has been identified as a major threat to biodiversity in aquatic ecosystems worldwide^25,26^. Freshwater ecosystems in particular are vulnerable to multiple stressors and severe impacts on their biodiversity have been documented^27–29^. For example, the proportion of cyprinid species in fish communities in north European lakes has increased and that change has been attributed to eutrophication^30^. Changes have also been documented in boreal waterbird communities at species level. Lehikoinen *et al*.^31^ found that populations of three of five waterbird species breeding in a wide range of habitats exhibited significant negative long-term trends in eutrophic but not in oligotrophic wetlands in Finland. In this case, southern populations in particular showed declining trends. The authors suggested that this was due to the environmental change caused by eutrophication, which has been more prevalent in southern Finnish lakes (exposed to both intensified agriculture and forestry)^32,33^ than in northern ones (affected mainly by forestry). Hence, we may expect long-term changes in the diversity of waterbird communities in boreal lakes, both in terms of latitudinal gradient and trophic status of lakes.

In this paper we study changes in species richness (alpha diversity) and species turnover (beta diversity; i.e. changes in community composition over time)^14^ of waterbird communities at the local (lake) level between two time periods, 1990/1991 and 2016. Assuming that population level changes translate into community level responses, we formulated two hypotheses. First, based on the finding that southern waterbird populations in particular have decreased in Finland^31^, we hypothesize that species richness has decreased more and species turnover has been higher, in southern than in northern waterbird communities. Second, because population declines have been found to be stronger in eutrophic lakes than in oligotrophic lakes^31,34^, we hypothesize that species richness has decreased more, and species turnover has been higher, in eutrophic lakes than in oligotrophic lakes.

Luxuriance and extent of aquatic emergent vegetation in lakes depend on trophic status (including anthropogenic eutrophication), species richness and abundance of helophytes (e.g. *Carex* spp., *Phragmites australis*, and *Typha latifolia*), and floating-leaved vegetation (e.g. *Nuphar lutea*) being highest in eutrophic and hypertrophic lakes^35^. While indicating lake trophic status, luxuriance and extent of these vegetation types also largely determine species richness and composition of boreal breeding waterbird communities^36,37^. Based on the luxuriance of helophyte and floating-leaved vegetation, Elmberg *et al*.^38^ developed a habitat structure index, which we used here as a measure of lake trophic status (see Material and methods). In addition, because metrics based on species’ ecological and functional traits are often more sensitive to environmental change than e.g. species richness *per se*, and hence may reveal additional information about drivers of biodiversity change^15^, we also studied differences in species richness within foraging guilds in local waterbird communities between 1990/1991 and 2016. We here focus on foraging guilds rather than functional groups^39,40^, because earlier findings suggest that foraging conditions in particular have changed in boreal lakes with impacts on waterbirds^31,41,42^. For example, assuming that eutrophication has caused changes in fish communities in boreal lakes (see above), we may expect divergent responses in piscivorous *versus* herbivorous waterbird species, as has been found in waterbird assemblages in winter^43^.

## Results

### Changes at community and guild levels

**Figure 1:**
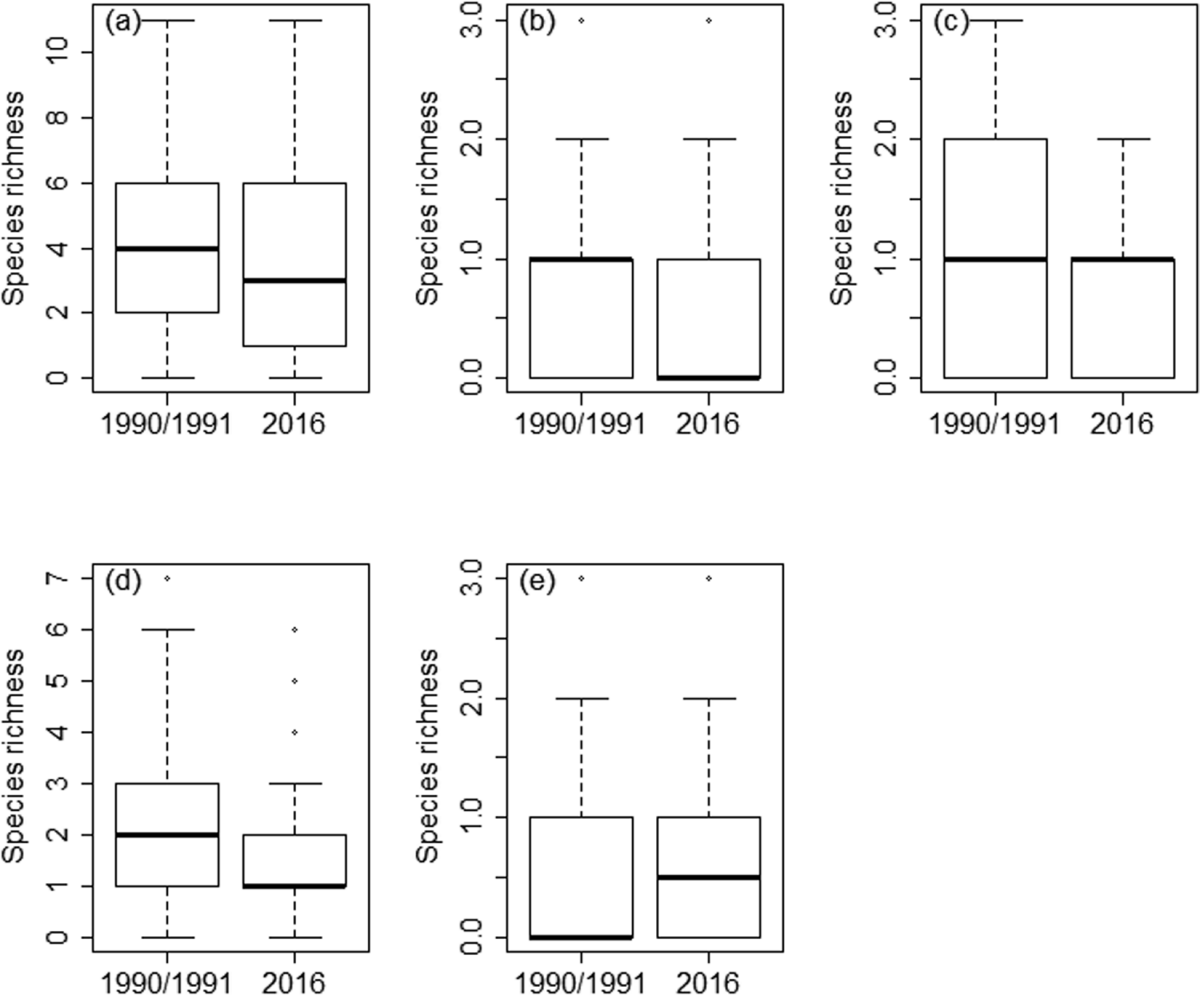
Box plots of total species richness (**a**) and species richness in four foraging guilds: piscivores (**b**), diving ducks (**c**), surface feeding waterbirds (**d**), and large herbivores (**e**) in local waterbird communities in 1990/1991 and in 2016. Mean ± SE are given; n = 58.

In general, species richness in local communities was lower in 2016 than in 1990/1991, although the difference was not quite significant when controlling for multiple comparisons (Fig. 1a; Wilcoxon signed-rank test, Z = −2.263, p = 0.024; the critical p*-*value using the B-Y method is p = 0.0219). Species turnover rate was relatively high (mean 48.7%), the number of species gained in local communities ranging between 0 and 5 (mean = 1.3) and the number of species lost between 0 and 6 (mean = 2.0). When the four foraging guilds were analysed separately, opposing differences in change of species richness were observed. Species richness of diving ducks (Fig. 1c; Wilcoxon signed-rank test, Z = −2.971, p = 0.003) and surface feeding waterbirds (Fig. 1d; Wilcoxon signed-rank test, Z = −2.539, p = 0.011) was lower in 2016 than in 1990/1991, whereas the species richness of large herbivores was higher (Fig. 1e; Wilcoxon signed-rank test, Z = 2.439, p = 0.015). No significant difference was found in the species richness of piscivores between the two study periods (Fig. 1b; Wilcoxon signed-rank test, Z = −1.603, p = 0.109).

### Contribution of guilds to community level changes

The contribution of guild level changes in species richness to changes in the four community characteristics varied depending on the community characteristics. Not surprisingly, because change in species richness at community level is the sum of changes in species richness within the four foraging guilds, all the guilds contributed significantly to change in species richness, the relative contribution (based on AIC values) being greatest for surface feeding waterbirds and smallest for large herbivores (Table 1). Guild level changes in species richness of the four guilds were not associated with species turnover rate (community level) (Tables 1 and 2). Number of species gained (community level) was explained by changes (increase) in the species richness of large herbivores (sum of the w_i_: 1.000) and surface feeding waterbirds (sum of the w_i_: 0.945), whereas the contribution of changes in the species richness of piscivores and diving ducks was not significant (Tables 1 and 2). Finally, changes (decrease) in species richness of piscivores, diving ducks and surface feeding waterbirds (sum of the w_i_ for all guilds: 1.000) contributed to the number of species lost (community level) while change in species richness of large herbivores did not (Tables 1 and 2).

**Table 1:** Models of guild level changes in species richness used to explain change at community level in species richness, species turnover rate, number of species gained, and number of species lost from 1990/1991 to 2016 in local (lake level) waterbird communities.

Model	β	SE	t	p	AIC_c_
**Change in species richness**					
Piscivores	1.687	0.319	5.292	<0.001	234.5
Diving ducks	1.288	0.314	4.104	<0.001	242.9
Surface feeding waterbirds	1.151	0.140	8.198	<0.001	212.4
Large herbivores	1.141	0.322	3.546	<0.001	246.1
**Model**		**k**	**AIC**_**c**_	**ΔAIC**_**c**_	**w**_**i**_
**Species turnover rate**					
Surface feeding waterbirds		4	24.5	0.00	0.222
Null model (intercept only)		3	24.7	0.16	0.205
**Number of species gained**					
Surface feeding waterbirds + Large herbivores		5	155.2	0.00	0.418
Surface feeding waterbirds + Large herbivores + Piscivores		6	156.1	0.95	0.261
Surface feeding waterbirds + Large herbivores + Diving ducks		6	156.9	1.76	0.174
Null model (intercept only)		3	189.0	33.86	0.000
**Number of species lost**					
Piscivores + Diving ducks + Surface feeding waterbirds		6	161.6	0.00	0.772
Null model (intercept only)		3	236.4	74.82	0.000

Because change in species richness at community level is the sum of guild level changes in species richness, a global model including all the guilds was not feasible. Therefore, separate models including only one guild were fitted for change in species richness (parameter estimate (β) and its standard error together with test statistics are presented for each model). For species turnover rate, number of species gained, and number of species lost, only models with ΔAIC_c_ ≤ 2 (ΔAIC_c_ = AIC_ci_ − AIC_cmin_) are presented together with the null model (see Material and methods).

**Table 2:** Model-averaged parameter estimates (β-values) and their 95% confidence intervals for guild level changes in species richness used to explain change in species turnover rate, number of species gained, and number of species lost from 1990/1991 to 2016 in local (lake level) waterbird communities.

Predictor	β	95% confidence interval	
		Lower	Upper
**Species turnover rate**			
Piscivores	0.02	−0.08	0.12
Diving ducks	0.01	−0.08	0.10
Surface feeding waterbirds	0.04	−0.01	0.09
Large herbivores	0.02	−0.07	0.11
**Number of species gained**			
Piscivores	−0.21	−0.53	0.11
Diving ducks	−0.12	−0.40	0.16
Surface feeding waterbirds	−0.25	−0.42	−0.09
Large herbivores	−0.94	−1.22	−0.67
**Number of species lost**			
Piscivores	0.84	0.51	1.16
Diving ducks	0.91	0.62	1.20
Surface feeding waterbirds	0.76	0.59	0.93
Large herbivores	0.06	−0.23	0.34

### Community-specific changes in relation to latitude and habitat index

With respect to change in species richness and species turnover rate between 1990/1991 and 2016 in relation to latitude and habitat, none of the models including one or more predictors fitted the data better than the null model (i.e. the null model was among the top models with ΔAIC_c_ ≤ 2; Table 3). This implies that habitat index and latitude were not good predictors of these community characteristics (Table 4). Nor were latitude and habitat index good predictors of changes in species richness and species turnover rate between 1990/1991 and 2016 within the four foraging guilds (Supplementary Table S1), with two exceptions worth mentioning. The models including latitude (see Supplementary Table S1) indicate that species richness of surface feeding waterbirds decreased from 1990/1991 to 2016 more in northern communities than in southern communities, whereas species richness of large herbivores increased more from 1990/1991 to 2016 in northern communities (Supplementary Table S2), although the model including latitude did not fit data better than the null model for the latter guild (Supplementary Table S1).

**Table 3:** Models used to explain change in species richness, species turnover rate, number of species gained, and number of species lost from 1990/1991 to 2016 in local (lake level) waterbird communities.

Model	k	AIC_c_	ΔAIC_c_	w_i_
**Change in species richness**				
Lake size	4	254.6	0.00	0.378
Null model (intercept only)	3	256.2	1.67	0.164
Lake size + Latitude	5	256.4	1.80	0.154
**Species turnover rate**				
Null model (intercept only)	3	24.7	0.00	0.377
Habitat index	4	26.4	1.69	0.162
**Number of species gained**				
Habitat index	3	169.3	0.00	0.340
Habitat index + Lake size	4	170.3	1.00	0.207
Habitat index + Latitude	4	171.0	1.70	0.146
Null model (intercept only)	2	174.4	5.13	0.026
**Number of species lost**				
Lake size	3	209.4	0.00	0.283
Lake size + Latitude	4	209.6	0.14	0.264
Lake size + Habitat index	4	209.6	0.23	0.252
Lake size + Latitude + Habitat index	5	210.2	0.76	0.193
Null model (intercept only)	2	227.3	17.94	0.000

Only models with ΔAIC_c_ ≤ 2 (ΔAIC_c_ = AIC_ci_ − AIC_cmin_) are presented together with the null model (see Material and methods).

**Table 4:** Model-averaged parameter estimates (β-values) and their 95% confidence intervals for predictor variables used to explain change in species richness, species turnover rate, number of species gained, and number of species lost from 1990/1991 to 2016 in local (lake level) waterbird communities.

Predictor	β	95% confidence interval	
		Lower	Upper
**Change in species richness**			
Habitat index	0.05	−0.58	0.67
Lake size	0.55	0.02	1.09
Latitude	0.19	−0.42	0.80
**Species turnover rate**			
Habitat index	−0.03	−0.11	0.04
Lake size	0.02	−0.05	0.10
Latitude	0.03	−0.11	0.17
**Number of species gained**			
Habitat index	0.30	0.05	0.56
Lake size	0.19	−0.05	0.43
Latitude	0.09	−0.14	0.33
**Number of species lost**			
Habitat index	0.18	−0.07	0.44
Lake size	0.36	0.18	0.55
Latitude	0.16	−0.05	0.37

The top models for number of species gained and number of species lost did not include the null model (Table 3). Habitat index was included in all the top models for number of species gained, whereas lake size and latitude only occurred in one of the models. The relative importance of predictors (sum of the w_i_) was 0.792 for habitat index, 0.479 for lake size, and 0.306 for latitude. The association between habitat index and number of species gained indicated that lakes with rich vegetation gained more species than lakes with sparse vegetation (Table 4). As to number of species lost, lake size occurred in all the top models, whereas habitat index and latitude occurred in two. Lake size was the most important predictor of number of species lost (sums of the w_i_: lake size 0.992, latitude 0.460, habitat index 0.453); number of species lost increased with lake size (Table 4). All in all, latitude appeared not to play any role in affecting changes in community characteristics, whereas habitat index was associated with changes in community composition, in particular the number of species gained.

## Discussion

We studied changes in species richness and composition of local boreal waterbird communities between two time periods, 1990/1991 and 2016. The lakes inhabited by the waterbird communities represented not only local trophic gradients but also a wide latitudinal gradient within the boreal biome. We found that, while species richness in local waterbird communities tended to be lower in 2016 than in 1990/1991, species turnover rate was relatively high, and both the number of species lost and the number of species gained varied considerably among communities. In addition, different foraging guilds exhibited contrasting changes; while the number of diving duck and surface feeding species was lower in 2016 than in 1990/1991, the number of large herbivorous species increased. Temporal changes in community characteristics did not show any association with latitude. Nor did they show an association with the habitat index, except that the number of species gained in local communities was higher in lakes with rich vegetation than in lakes with sparse vegetation (see below).

By and large, the observed decrease in species richness in local waterbird communities is in line with global trends of overall decrease in biological diversity^1–3^, including freshwater ecosystems^44^. At the same time, the high species turnover in local waterbird communities found here echoes recent calls to pay more attention to changes in the composition of local communities^7,8,13,45,46^. Similarly, the finding that three foraging guilds showed contrasting changes in species richness goes hand in hand with the recent emphasis^15^ on how the study of changes in functional and ecological community characteristics enhances the unravelling of the processes driving community change in response to environmental change. This view is supported also by our observation that changes in species richness within the four foraging guilds contributed differently to two community characteristics, the number of species gained and the number of species lost.

Historically, north European bird communities have been characterized by relatively high species turnover, as demonstrated by Järvinen and Ulfstrand^47^ for the period 1850 to 1970. Eutrophic lakes in particular have been colonized by several new species since the 19^th^ century^47,48^. According to more recent Finnish data^31,34^, many species and populations inhabiting eutrophic lakes are now in decline. This suggests that species richness in eutrophic lakes is also in decline. However, negative changes in breeding abundance do not seem to have occurred at the community level, as we did not find strong associations between the habitat index and changes in species richness and the number of species lost from local communities. On the contrary, the number of species gained increased with the habitat index score (a proxy for trophic status of the study lakes), suggesting that the trend of increasing species richness in eutrophic lakes is actually continuing^47,48^. The association between the number of species gained and the habitat index may be explained by higher food availability and a larger number of foraging microhabitats in lakes supporting rich vegetation. If eutrophication goes on, more dramatic changes at the community level are to be expected and local species richness of waterbirds may decrease in the future (see below). This is because current overall species richness of breeding waterfowl (order Anseriformes) in Europe peaks north of 60°N^49^, and marked northward shifts due to climate change are possible only for a few species that breed on eutrophic wetlands in central and southern Europe^50^.

Detailed species level considerations (e.g. which species are lost and which are gained) are out of the scope of the current study and will be addressed elsewhere (Elmberg *et al*., in preparation). Anyhow, our finding that different foraging guilds showed contrasting temporal change in species richness implies that ecological conditions in boreal lakes have changed. In particular, the number of species in the local foraging guilds that are fully or almost fully dependent on aquatic food webs (diving ducks and surface feeding waterbirds) was lower in 2016 than in 1990/1991 (although the corresponding difference was not significant in piscivores), whereas the number of large herbivores (swans and geese), which use terrestrial habitats too for foraging, have increased in 2016 compared to 1990/1991. These findings support the hypothesis that foraging conditions for waterbirds in many boreal lakes have deteriorated due to anthropogenic impacts, as has been previously suggested for declining diving waterbirds in eutrophic lakes^31,34,51^. As to the increase of large herbivores, it is possible that conditions in wintering areas have improved (e.g. food limitation has decreased or ceased), augmenting numbers of breeding birds in boreal lakes. It is perhaps worth noting that the change in species richness of large herbivores was not associated with the habitat index, suggesting that large herbivores have increased in all types of lakes. On the other hand, habitat index did not explain changes in species richness and species turnover rate in the other guilds either. In general, because the number of species within the four foraging guilds is rather small (range 4–9), one should be cautious when making conclusions about factors that could explain guild level changes in species richness and species turnover.

The number of species gained increased with the lake-specific habitat index. Because overall change in species richness was not associated with the habitat index, compensatory changes in the composition of local communities had probably taken place, although the number of species lost did not increase strongly with the habitat index. Moreover, the contrasting changes of species number among foraging guilds suggest that compensatory changes have occurred. Interestingly, it has been found in lakes in southern Sweden that, while naturally eutrophic lakes supported more species than oligotrophic, anthropogenic eutrophication did not lead to higher species richness in formerly oligotrophic lakes^52^. The first finding of that study is in line with our assertion that species richness increases with lake trophic status as measured by the habitat index (see Material and methods and Supplementary Fig. S1). Because that study did not investigate temporal change in species richness and community composition, it remains unknown if compensatory changes in species composition had taken place in those lakes that were subject to anthropogenic eutrophication. Nevertheless, their finding that human-caused eutrophication did not increase species richness suggests there is a threshold above which further eutrophication will not increase species richness. We do not know whether the trophic status of our study lakes has changed between 1990/1991 and 2016. Hence, we cannot say whether the differences in community characteristics observed in the present are due to a possibly continuing eutrophication or other anthropogenic stressors, many of which are associated with global drivers such as climate change^53,54^. We know, however, that previously large stands of *Equisetum fluviatile*, a vegetation type that contributes to our habitat index^38^, have decreased in the study lakes^55^, implying that habitat change in them has occurred over the time span of this study.

It was surprising to us that temporal changes in community characteristics did not show any clear pattern along the south-north gradient, in particular considering the fact that shifts in range and mean distribution along this gradient have been documented for several bird species breeding in northern Europe^23,24^. One explanation for the absence of such a latitudinal trend is that possible shifts in range or distribution have been compensatory among species. Unfortunately, previous studies^23,24^ did not consider shifts in individual waterbird species so we cannot say if compensatory shifts have occurred or not. However, because shifts in breeding distribution are typically only a few kilometers per year^24^ (although that work considered mostly Passerines), and the south-north gradient covered in our study extends well over 1 000 km, we do not believe compensatory shifts have had major impact on the results. If any such effects occurred, they were probably overridden by local factors, such as wetland trophic status. Moreover, even though large scale population trends may suggest contrasting colonization-extinction dynamics among species, such dynamics may not be realized at the level of the local community^56^.

Because our data are snapshots from two time periods 25 years apart, stochasticity may have affected the results. However, we do not believe this is the case, partly because the changes in total species richness and guild species richness found here are in line with species-specific population trends based on the long-term waterbird monitoring data from Finland^31,34^ (Elmberg *et al*., in preparation). For example, the percentage changes in the number of lakes occupied in 2016 compared to that in 1990/1991 (see Supplementary Table S3) are correlated with annual population growth rates in Finland in 1986–2013 among 16 waterbird species occurring in the waterbird communities studied here (see Supplementary Fig. S2). It is also noteworthy in this context that the number of species lost increased with lake size. Considering that population sizes of waterbirds generally increase with lake size^57,58^, this finding suggests that stochastic local extinctions due to small population size alone do not explain the number of species lost and hence the decrease of species richness in our data. Finally, it is unlikely that possible changes in detection probability of individual species could explain the changes in guild and community level characteristics between 1990/1991 and 2016 (see Supplementary Appendix S1).

In conclusion, species richness of local waterbird communities of boreal lakes was lower in 2016 than in 1990/1991, but changes in their composition were even more pronounced. In particular, species turnover rate turned out to be high, and changes in the number of species between the two time periods showed opposite patterns among different foraging guilds. We suggest that understanding changes in biodiversity of local communities in boreal lakes would benefit from simultaneous consideration of local processes and their large-scale drivers.

## Material and Methods

### Bird data

We repeated waterbird surveys carried out in a previous study^38^ in which ten lakes were selected in each of six study regions (i.e. in all 60 lakes) between 56° and 67°N in Finland and Sweden (see Supplementary Table S4), to represent local gradients from eutrophic to oligotrophic conditions as indicated by the luxuriance of aquatic vegetation^38^ (see also below). On each of the 60 lakes, waterbirds were counted in either 1990 or 1991 according to the methods described in our earlier study^38^. In brief, two surveys of settling waterbird pairs were done in April and May using the point count method^59^. The timing of surveys took into account differences in spring phenology among the regions (see Supplementary Table S4). We repeated waterbird surveys in 2016 on the same lakes using the same method and field protocol as in our earlier study^38^, with the following exceptions. Point counts were done only once in 2016 in region 1 (see Fig. 1 in the earlier study)^38^. The date of this single count was approximately in the middle between the dates of the two counts in 1990/1991. In addition, two lakes were subsequently excluded from the analyses; one from region 1 due to increased human settlement (i.e. disturbance), and one from region 3, which did not have any bird observations in either study period. Consequently, the final sample size in the present study was 58 local communities. Waterbird observations were interpreted as pair numbers using species-specific criteria^59^; as in our earlier study^56^, a species was considered to be present in a community in a given year if at least one breeding pair was observed in either of the two surveys. For further information on waterbird surveys and data, see Supplementary Appendix S1.

### Diversity measures

When considering temporal changes in diversity, a general recommendation is to use more than one diversity index, as index choice may affect results^60,61^. We considered four community characteristics that describe changes in species richness and composition in local communities from 1990/1991 to 2016: 1) change in species richness, 2) number of species gained, 3) number of species lost, and 4) species turnover rate. We calculated species turnover rate in local communities between 1990/1991 and 2016 in percent of the species pool^62^: turnover rate = 100 × [(E + H)/(C + D)], where C is the number of species in 1990/1991, D is the number of species in 2016, E is the number of species present in 1990/1991 but not in 2016, and H is the number of species present in 2016 but not in 1990/1991. In addition to these community characteristics, we considered change (between 1990/1991 and 2016) in the number of species in four waterbird foraging guilds: piscivores, diving ducks, surface feeding waterbirds, and large herbivores. Each species was assigned to one of the four guilds according to its principal foraging habit (see Supplementary Table S3).

### Habitat index

We used a habitat structure index developed in our earlier study, based on the abundance of helophyte and floating-leaved vegetation^38^. In brief, we mapped vegetation in each of the 60 original study lakes in July 1990/1991. We measured vegetation heterogeneity, the cover of floating vegetation, and the taxonomic composition, width and height of emergent shoreline vegetation (18 variables in all), and then used principal component analysis to derive composite gradients of habitat structure along which the 60 lakes were placed. The first principal component axis represented a gradient from lakes with low and narrow belts of sparse emergent vegetation (high negative scores on 1^st^ axis) to lakes with tall, wide and heterogeneous emergent and abundant floating vegetation (high positive scores on 1^st^ axis) (see the original study^38^ for details). Lakes with a high positive score typically had large stands of *Phragmites australis*, *Equisetum fluviatile*, *Typha latifolia*, *Scirpus lacustris*, and *Carex* spp., whereas lakes with a high negative score instead had shores that were either stony or lined by bogs or open fens (typically floating *Sphagnum*). We used each lake’s value on the 1^st^ axis as an index (hereafter, ‘habitat index’) of trophic status. This is biologically meaningful to use for studies of local waterbird communities, as exemplified by the finding that the number of species in local dabbling duck guilds correlated positively with this habitat index^38^. Similarly, total species richness of local waterbird communities in the 1990/1991 data set correlated positively with the habitat index (multiple regression controlling for a lake size effect; habitat index, β = 0.429, SE = 0.112, t = 3.836, p < 0.001; lake size, β = 0.349, SE = 0.112, t = 3.119, p = 0.003; analysis based on values standardized within regions; for a plot based on original values, see Supplementary Fig. S1). Moreover, the correlation between total species richness and the habitat index was strong also in the 2016 bird data (Supplementary Fig. S1), implying a robust association between species richness and habitat complexity in our data set.

### Statistical analyses

We used the Wilcoxon matched-pairs signed-rank test to compare overall changes in species richness at community level and within foraging guilds between the two time periods, 1990/1991 and 2016. We used the false discovery rate method developed by Benjamini and Yekutieli^63^ (hereafter the B-Y method) to control for multiple comparisons. Narum^64^ gives critical values for the B-Y method for multiple tests ranging from 1 to 100. The number of multiple comparisons in our study was five; hence, the critical p-value using the B-Y method is p = 0.0219. In Results we present unadjusted p-values and refer to the B-Y adjusted p-value when assessing statistical significance.

We used generalized linear mixed-effects models to assess the contribution of guild level changes in species richness to temporal changes in community characteristics and if temporal changes in community characteristics were associated with latitude and habitat index. Because change in species richness is the sum of guild level changes in species richness, a global model including all guilds was not feasible; therefore, we fitted separate models including only one guild when assessing the contribution of guild level changes in species richness to community level change in species richness. As the habitat index was correlated with lake size, and lake size in turn correlated with temporal change in some of the community characteristics (see Supplementary Table S5), we included also lake size as a covariate. Lake-specific latitudes were included as decimal degrees. To check for multicollinearity we calculated the variance inflation factor (VIF) for each predictor by doing a linear regression with the predictor of interest as the dependent variable and the other predictors as explanatory variables; using the *r*^2^ of that regression, VIF = 1/(1 − *r*^2^). Multicollinearity is generally considered a problem if VIF > 2.50. This was not the case with the predictors of our study: latitude, VIF = 1.003; habitat index, VIF = 1.043; lake size, VIF = 1.047. We used standardized (z-scores) values of the predictor variables to facilitate comparisons of β-values^65^. To control for potential non-independence of lake-specific data at the regional level, we included region as a random factor in the models.

For change in species richness and species turnover rate, we used lme function from the package nlme^66^; normality of residuals was tested with the Shapiro-Wilk test and was found to be so for both response variables. For number of species gained and number of species lost we assumed a Poisson distribution and made the analysis with the glmer function from the package lme4^67^. Analyses were performed using program R 3.4.0^68^. We fitted all possible models (i; in total eight models, including a model containing only an intercept, i.e. the ‘null’ model) to the data for each dependent variable (i.e. change in species richness, species turnover rate, number of species gained, and number of species lost) and used Akaike’s information criterion corrected for small sample size (AIC_c_) to assess model fit^69^. Specifically, differences in AIC_c_ (i.e. ΔAIC_c_ = AIC_ci_ − AIC_cmin_) and model-specific weights (Akaike weights, w_i_) were used to assess model fit; models with ΔAIC_c_ ≤ 2 have substantial support, whereas models where ΔAIC_c_ is greater have progressively less support^69^. We used the ΔAIC_c_ ≤ 2 criterion for presenting competitive models, i.e. top models. As there was uncertainty in model selection (i.e. several competitive models; see Results), we calculated unconditional model-averaged parameter values (β-values) and their 95% confidence intervals (CI) for the predictor variables using all the models^69^. Inference about the importance of predictor variables was based on the β-values (and 95% confidence intervals, i.e. ‘significant’ if the interval did not include 0) and the sum of the *w*_*i*_ that was calculated over all models for each predictor^69^.

## Supplementary information

Changes in species richness and composition of boreal waterbird communities: a comparison between two time periods 25 years apart

## Data Availability

The datasets generated and analysed in the current study are included in its Supplementary Information files or are available from the corresponding author on reasonable request.
