# Supplementary material for: Changes in species richness and composition of boreal waterbird communities: a comparison between two time periods 25 years apart
Source: Sci Rep. 2019 Feb 11;9:1725. doi: 10.1038/s41598-018-38167-1 (PMC6370776; doi:10.1038/s41598-018-38167-1)
Supplement: Supplementary file 1 — Changes in species richness and composition of boreal waterbird communities: a comparison between two time periods 25 years apart [file 41598_2018_38167_MOESM1_ESM.docx]

**Supplementary Information to:**

**Changes in species richness and composition of boreal waterbird communities: a comparison between two time periods 25 years apart**

Hannu Pöysä^1*^, Sari Holopainen^2^, Johan Elmberg^3^, Gunnar Gunnarsson^3^, Petri Nummi^2^ & Kjell Sjöberg^4^

^1^ Natural Resources, Natural Resources Institute Finland (Luke), Joensuu, Finland

^2^ Department of Forest Sciences, University of Helsinki, Finland

^3^ Department of Environmental Science and Bioscience, Kristianstad University, Sweden

^4^ Department of Wildlife, Fish, and Environmental Studies, Swedish University of Agricultural Sciences, Umeå, Sweden

*Correspondence: Hannu Pöysä, Natural Resources, Natural Resources Institute Finland (Luke), Yliopistokatu 6, FI-80100 Joensuu, Finland

E-mail: hannu.poysa@luke.fi

Phone: +358 400 103 081

ORCID: 0000-0002-1585-2375

Supplementary Table S1. Models used to explain guild level changes in species richness and species turnover rate from 1990/1991 to 2016 in local (lake level) waterbird communities. Only models with ΔAIC_c_ ≤ 2 (ΔAIC_c_ = AIC_ci_ - AIC_cmin_) are presented together with the null model (see Material and methods in the main article).

| Model | k | AIC_c_ | ΔAIC_c_ | w_i_ |
| --- | --- | --- | --- | --- |
| Piscivores: Change in species richness |  |  |  |  |
| Null model (intercept only) | 3 | 96.4 | 0.00 | 0.463 |
|  |  |  |  |  |
| Piscivores: Species turnover rate |  |  |  |  |
| Habitat index | 4 | 46.3 | 0.00 | 0.334 |
| Null model (intercept only) | 3 | 46.7 | 0.45 | 0.266 |
|  |  |  |  |  |
| Diving ducks: Change in species richness |  |  |  |  |
| Null model (intercept only) | 3 | 120.3 | 0.00 | 0.294 |
| Lake size | 4 | 120.8 | 0.46 | 0.234 |
| Latitude | 4 | 121.7 | 1.35 | 0.150 |
|  |  |  |  |  |
| Diving ducks: Species turnover rate |  |  |  |  |
| Null model (intercept only) | 3 | 57.5 | 0.00 | 0.284 |
| Habitat index | 4 | 58.0 | 0.52 | 0.219 |
| Habitat index + Latitude | 5 | 59.1 | 1.62 | 0.126 |
| Latitude | 4 | 59.3 | 1.76 | 0.117 |
| Lake size | 4 | 59.4 | 1.93 | 0.108 |
|  |  |  |  |  |
| Surface feeding waterbirds: Change in species richness |  |  |  |  |
| Lake size + Latitude | 5 | 196.4 | 0.00 | 0.514 |
| Null model (intercept only) | 3 | 202.4 | 5.97 | 0.026 |
|  |  |  |  |  |
| Surface feeding waterbirds: Species turnover rate |  |  |  |  |
| Null model (intercept only) | 3 | 46.5 | 0.00 | 0.364 |
| Latitude | 4 | 47.6 | 1.17 | 0.203 |
|  |  |  |  |  |
| Large herbivores: Change in species richness |  |  |  |  |
| Null model (intercept only) | 3 | 90.9 | 0.00 | 0.279 |
| Latitude | 4 | 90.9 | 0.01 | 0.278 |
| Lake size | 4 | 92.2 | 1.37 | 0.141 |
|  |  |  |  |  |
| Large herbivores: Species turnover rate |  |  |  |  |
| Null model (intercept only) | 3 | 39 | 0.00 | 0.282 |
| Habitat index | 4 | 39.0 | 0.04 | 0.276 |
| Lake size | 4 | 40.6 | 1.67 | 0.122 |

Supplementary Table S2. Model-averaged parameter estimates (β-values) and their 95% confidence intervals for predictor variables used to explain guild level changes in species richness and species turnover from 1990/1991 to 2016 in local (lake level) waterbird communities.

|  | | 95% confidence interval | |
| --- | --- | --- | --- |
| Predictor | β | Lower | Upper |
| Piscivores: Change in species richness |  |  |  |
| Habitat index | 0.05 | -0.24 | 0.33 |
| Lake size | 0.09 | -0.2 | 0.39 |
| Latitude | 0 | -0.41 | 0.41 |
|  |  |  |  |
| Piscivores: Species turnover rate |  |  |  |
| Habitat index | -012 | -0.25 | 0.01 |
| Lake size | 0.01 | -0.14 | 0.17 |
| Latitude | 0.04 | -0.11 | 0.19 |
|  |  |  |  |
| Diving ducks: Change in species richness |  |  |  |
| Habitat index | -0.01 | -0.29 | 0.27 |
| Lake size | 0.17 | -0.07 | 0.41 |
| Latitude | -0.12 | -0.37 | 0.13 |
|  |  |  |  |
| Diving ducks: Species turnover rate |  |  |  |
| Habitat index | -0.09 | -0.22 | 0.03 |
| Lake size | -0.03 | -0.16 | 0.09 |
| Latitude | -0.06 | -0.19 | 0.06 |
|  |  |  |  |
| Surface feeding waterbirds: Change in species richness |  |  |  |
| Habitat index | 0.21 | -0.18 | 0.61 |
| Lake size | 0.41 | 0.07 | 0.75 |
| Latitude | 0.46 | 0.12 | 0.80 |
|  |  |  |  |
| Surface feeding waterbirds: Species turnover rate |  |  |  |
| Habitat index | -0.01 | -0.10 | 0.09 |
| Lake size | 0.02 | -0.07 | 0.11 |
| Latitude | 0.10 | -0.07 | 0.26 |
|  |  |  |  |
| Large herbivores: Change in species richness |  |  |  |
| Habitat index | 0.13 | -0.33 | 0.59 |
| Lake size | 0.17 | -0.15 | 0.49 |
| Latitude | -0.38 | -0.72 | -0.03 |
|  |  |  |  |
| Large herbivores: Species turnover rate |  |  |  |
| Habitat index | 0.15 | -0.03 | 0.34 |
| Lake size | 0.06 | -0.08 | 0.20 |
| Latitude | 0.07 | -0.09 | 0.22 |


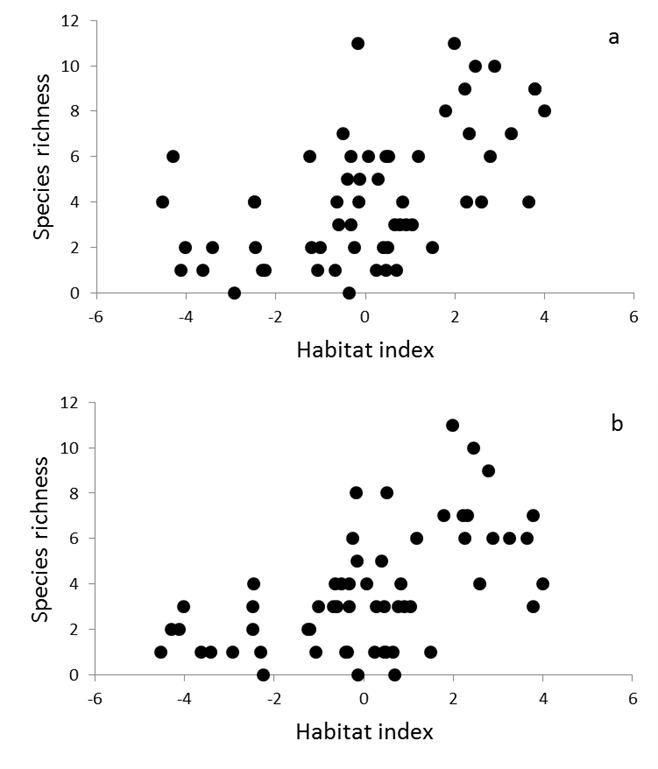


Supplementary Figure S1. Relationship between total species richness in local (lake level) waterbird communities and habitat index in 1990/1991 (a; r = 0.545, p < 0.001, n = 58) and 2016 (b; r = 0.559, p < 0.001, n = 58).

Supplementary Table S3. Species of local waterbird communities studied (in alphabetical order). For each species is given foraging guild (piscivore, diving duck, surface feeding waterbird, and large herbivore), body mass^3^, habitat use index, number of lakes occupied in 1990/1991, number of lakes occupied in 2016, and percentage change in the number of lakes occupied (i.e. from 1990/1991 to 2016; change in lake occupation rate is in parentheses for species that had zero occupation in 1990/1991, i.e. division by zero). Foraging guild classification was based on principal foraging habit^4-6^. Habitat use index is the mean of the habitat index values (see Material and methods, Habitat index, in the main text) of the lakes in which the species occurred either in 1990/1991 or in 2016. The species that were included to study the relationship between ‘Percentage change in lake occupation rate’ and population growth rate in Finland in 1986–2013 are denoted by an asterisk (*).

| Species | Foraging guild | Body mass (g) | Habitat use index | Number of lakes occupied in 1990/1991 | Number of lakes occupied in 2016 | Percentage change in lake occupation rate |
| --- | --- | --- | --- | --- | --- | --- |
| *Anas acuta** | Surface feeding | 740 | 1.31 | 7 | 3 | -57.1 |
| *Anas clypeata** | Surface feeding | 600 | 2.16 | 7 | 6 | -14.3 |
| *Anas crecca** | Surface feeding | 300 | 0.30 | 30 | 26 | -13.3 |
| *Anas platyrhynchos** | Surface feeding | 1 100 | 0.19 | 47 | 42 | -10.6 |
| *Anas querquedula** | Surface feeding | 365 | 2.26 | 7 | 2 | -71.4 |
| *Anas strepera* | Surface feeding | 755 | 3.55 | 1 | 3 | 200.0 |
| *Anser anser* | Herbivore | 3 510 | 1.85 | 4 | 11 | 175.0 |
| *Aythya ferina** | Diving duck | 870 | 1.90 | 4 | 0 | -100.0 |
| *Aythya fuligula** | Diving duck | 720 | 1.11 | 20 | 10 | -50.0 |
| *Branta canadensis* | Herbivore | 4 350 | 1.00 | 7 | 9 | 28.6 |
| *Branta leucopsis* | Herbivore | 1 720 | 2.78 | 0 | 1 | (100.0) |
| *Bucephala clangula** | Diving duck | 745 | 0.22 | 35 | 31 | -11.4 |
| *Cygnus cygnus* | Herbivore | 9 050 | 1.32 | 3 | 16 | 433.3 |
| *Cygnus olor* | Herbivore | 11 000 | 1.87 | 9 | 2 | -77.8 |
| *Fulica atra** | Surface feeding | 520 | 1.93 | 9 | 8 | -11.1 |
| *Gavia arctica** | Piscivore | 2 350 | -1.41 | 5 | 8 | 60.0 |
| *Gavia stellata* | Piscivore | 1 630 | 2.72 | 0 | 3 | (100.0) |
| *Mareca penelope** | Surface feeding | 680 | 0.78 | 18 | 9 | -50.0 |
| *Mergellus albellus* | Diving duck | 545 | -0.32 | 1 | 0 | -100.0 |
| *Mergus merganser** | Piscivore | 1 520 | -0.75 | 14 | 5 | -64.3 |
| *Mergus serrator** | Piscivore | 950 | 3.26 | 1 | 0 | -100.0 |
| *Podiceps auritus** | Piscivore | 560 | 1.52 | 4 | 1 | -75.00 |
| *Podiceps cristatus** | Piscivore | 995 | 2.03 | 11 | 7 | -36.4 |
| *Podiceps grisegena** | Piscivore | 845 | 1.97 | 5 | 7 | 40.0 |
| *Tadorna tadorna* | Surface feeding | 1 150 | 0.78 | 1 | 1 | 0.0 |


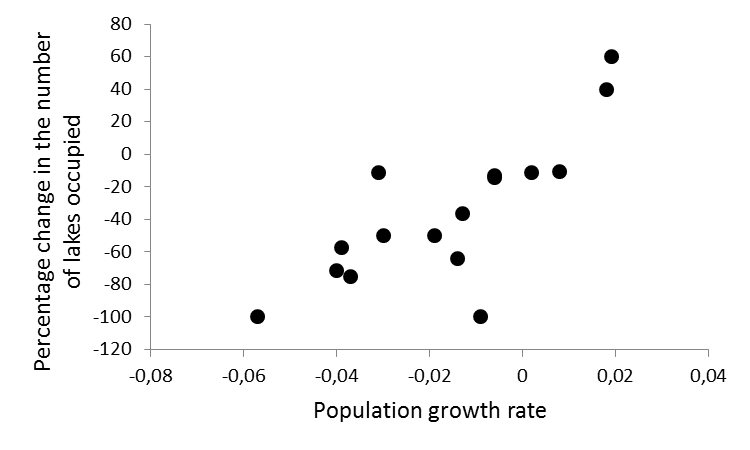


Supplementary Figure S2. Relationship between percentage change (from 1990/1991 to 2016) in the number of lakes occupied (this study, data from Table S3) and population annual growth rate in Finland in 1986–2013 (overall trends from Tables 2 and 3 in Lehikoinen et al.^1^ (r = 0.782, p < 0.001, n = 16 species). Population growth rates are based on annual data (see the original article for more details). Species included in the analysis are denoted by asterisks in Table S3.

Supplementary Appendix S1: **Detection probability and changes in community characteristics**

Could possible changes in detection probability of individual species explain the changes in guild and community level characteristics between 1990/1991 and 2016? We do not believe this is the case. First, we used the waterbird point count method^7^ in both periods and at all lakes. In each of study regions 2–6, the same person (one of us in each of the five study regions) did the point counts at all the 10 lakes both in 1990/1991 and in 2016; in study region 1 the counts were done by two experienced field assistants using the same standardized census protocol as we used (fixed census sites, same amount of time spent on each site, good weather conditions during the census, etc., i.e. following the field work guidelines specified in the manual^7^). The waterbird point count is a standardized method used for monitoring waterbirds in Finland. Censuses are done in late April and May, before the vegetation has started to grow, that is, visibility should be good. Koskimies & Pöysä^8^ tested the efficiency of the point count method to detect settled birds by comparing this method with round counts, which is an alternative waterbird census method^7^. In the round count, the same areas were censused by moving around the lake by a boat or by foot near the shoreline so that all the settled birds were detected with a high probability; the round count was done immediately after the point count. Koskimies & Pöysä found that, while total pair numbers obtained for different species differed somewhat between the point counts and the round counts, the methods gave almost identical results concerning species number, diversity, evenness and community composition. In addition, the community-level results held true even for lakes differing in size and type (barren oligotrophic lakes *versus* eutrophic lakes with rich vegetation). It is important to note that all the guild and community level metrics studied by us in the present study are based on species presence/absence data, not abundance data.

Second, considering the possibility that species-specific characteristics affect detection probability, we might expect that smaller bodied species in general have lower detection probability than larger bodied species and that species that breed on lakes with rich vegetation have lower detection probability than species that breed on lakes with sparse vegetation. To test if these possibilities could have affected the results of this study, we correlated the percentage change in the number of lakes occupied by the species (i.e. the number of lakes occupied in 2016 minus the number of lakes occupied in 1990/1991 divided by the number of lakes occupied in 1990/1991 times 100) with body size and habitat use index (data from Table S3; species *Branta leucopsis* and *Gavia stellata* were excluded due to insufficient data). The rationale here is that, if visibility has decreased from 1990/1991 to 2016 due to habitat change (but see below), smaller bodied species should have decreased more in terms of lake occupation rate than larger bodied species, and species that occur more frequently on lakes with rich vegetation (high positive habitat use index) should have decreased more in lake occupation rate than species that occur more frequently on lakes with sparse vegetation (high negative habitat use index). It turned out that smaller bodied species did not decrease more in lake occupation rate than larger bodied species, although the correlation was positive (Spearman rank correlation = 0.350, n = 23, p > 0.10; Figure S3a), nor did the change in lake occupation rate correlate with the species-specific habitat use index (Spearman rank correlation = -0.067, n = 23, p > 0.50; Figure S3b). The weak positive association between body mass and change in lake occupation rate was driven by the four largest species (*Cygnus cygnus*, *Anser anser*, *Gavia arctica*, and *Branta canadensis*; see Figure S3a); if these four species are excluded, the correlation is close to zero (Spearman rank correlation = 0.036, n = 19, p > 0.50). We would like to add that, because the four largest species with increased lake occupation rate are highly visible and easy to detect, it is unlikely that they were recorded as ‘absent’ in the 1990/1991 censuses due to low detection probability. Also note that *Cygnus cygnus*, *Anser anser,* and *Branta canadensis* are herbivores (see Table S3); their increase largely explains the increase of species richness of the large herbivores guild.

Third, as we have shown elsewhere^9^, and mention in the main text, previously wide stands of *Equisetum fluviatile* have decreased in the study lakes from 1990/1991 to 2013/2014. If anything, this habitat change has increased visibility, not decreased it. This is the only conspicuous difference in the richness of emergent vegetation (i.e. the vegetation type that potentially affects visibility) in the study lakes that we can tell between 1990/1991 and 2016 (see Suhonen et al.^10^ for additional information on long term habitat changes in boreal lakes).

Finally, it is noteworthy also in this context that there was a strong positive correlation between the percentage change in the number of lakes occupied (data from this study) and the annual population growth rate in Finland in 1986–2013 (data from Lehikoinen et al.^1^) among 16 species in the waterbird communities studied here (see Figure S2 and Table S3). Because changes in detection probability hardly can explain long-term trends in breeding numbers (annual population growth rates) in 1986–2013, this correlation suggests that the presence/absence data for the waterbird species in the present study are accurate enough and genuinely reflect changes in species abundances and occurrences.


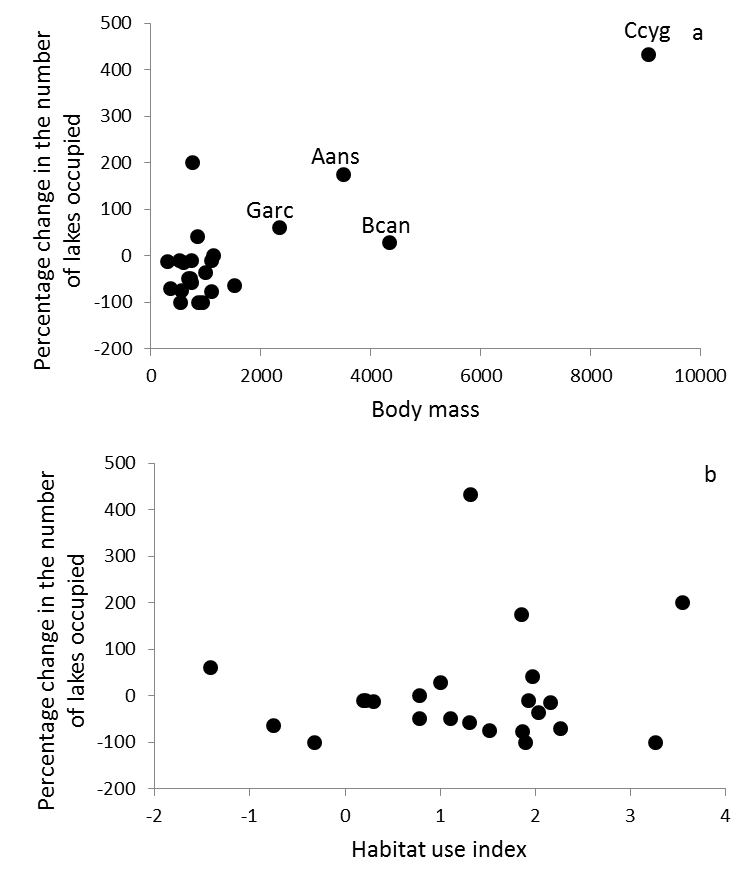


Supplementary Figure S3. Relationship between percentage change (from 1990/1991 to 2016) in the number of lakes occupied and (a) body mass and (b) habitat use index in waterbird species (n = 23 species; data are from Table S3).

Supplementary Table S4. Geographic coordinates of the southernmost, northernmost, westernmost and easternmost study lake in each of the six study regions in Finland and Sweden. The waterbird species recorded in the study regions and the timing of the first and the second waterbird censuses in 1990/1991 and 2016 are also given (only one census was done in 2016 in region 1). Region names and numbers correspond to those in Fig. 1 of Elmberg et al.^2^.

|  |  | Lappi (1), Finland | Västerbotten (2), Sweden | Häme (3), Finland | Karjala (4), Finland | Södermanland (5), Sweden | Scania (6), Sweden |
| --- | --- | --- | --- | --- | --- | --- | --- |
| Coordinates of furthermost lakes | South | 66°49’N | 63^o^45´N | 61°10’N | 61°32’N | 58^o^57´N | 55°53’N |
|  | North | 67°03’N | 64^o^02´N | 61°14’N | 61°39’N | 59^o^20´N | 56°19’N |
|  | West | 25°05’E | 19^o^36´E | 25°02’E | 29°33’E | 16^o^22´E | 14°12’E |
|  | East | 25°36’E | 20^o^22´E | 25°09’E | 29°47’E | 16^o^45´E | 14°24’E |
| Species recorded either in 1990/1991 or in 2016 | | *Anas acuta, Anas crecca, Anas platyrhynchos, Aythya fuligula, Bucephala clangula, Cygnus cygnus, Gavia arctica, Mergellus albellus, Mergus merganser* | *Anas acuta, Anas crecca, Anas platyrhynchos, Anas querquedula, Aythya fuligula, Branta canadensis, Branta leucopsis, Bucephala clangula, Cygnus cygnus, Fulica atra, Gavia arctica, Gavia stellate, Mareca penelope, Mergus merganser, Mergus serrator, Podiceps auritus, Podiceps cristatus, Podiceps grisegena* | *Anas crecca, Anas platyrhynchos, Aythya fuligula, Bucephala clangula, Cygnus cygnus, Fulica atra, Gavia arctica, Mareca penelope, Podiceps grisegena* | *Anas acuta, Anas clypeata, Anas crecca, Anas platyrhynchos, Anas querquedula, Aythya ferina, Aythya fuligula, Bucephala clangula, Cygnus cygnus, Fulica atra, Gavia arctica, Mareca penelope, Podiceps auritus, Podiceps cristatus, Podiceps grisegena* | *Anas clypeata, Anas crecca, Anas platyrhynchos, Anas strepera, Anas querquedula, Anser anser, Aythya ferina, Aythya fuligula, Branta canadensis, Bucephala clangula, Cygnus cygnus, Cygnus olor, Fulica atra, Gavia arctica, Mareca penelope, Podiceps cristatus* | *Anas clypeata, Anas crecca, Anas platyrhynchos, Anas querquedula, Anser anser, Aythya ferina, Aythya fuligula, Branta canadensis, Bucephala clangula, Cygnus olor, Fulica atra, Gavia arctica, Mareca penelope, Mergus merganser, Podiceps cristatus, Tadorna tadorna* |
| Dates of the first waterbird survey | 1990–1991 | 26 – 29 May | 14 May | 8 – 9 May | 27 April – 4 May | 29 April – 1 May | 9 – 10 April |
|  | 2016 | 20 May – 7 June | 12 May | 22 – 23 April | 25 – 28 April | 30 April – 2 May | 6 April |
| Dates of the second waterbird survey | 1990–1991 | 7 – 12 June | 28 May | 22 – 23 May | 15 – 18 May | 13 – 15 May | 2 – 3 May |
|  | 2016 |  | 28 May | 21 – 22 May | 1 – 6 May | 16 – 17 May | 4 May |

Supplementary Table S5. Pair-wise Pearson correlations between lake size, habitat index, and community characteristics based on non-standardized data. Significant (p < 0.05) correlations are given in bold; n = 58 in all cases. Lake size and habitat index data are from 1990/1991. The community characteristics included measure changes in species numbers and species turnover rate between 1990/1991 and 2016.

|  | Lake size | Habitat index | Change in species richness | Number of species gained | Number of species lost | Species turnover rate |
| --- | --- | --- | --- | --- | --- | --- |
| Lake size | 1.000 |  |  |  |  |  |
| Habitat index | **0.440** | 1.000 |  |  |  |  |
| Change in species richness | 0.255 | 0.060 | 1.000 |  |  |  |
| Number of species gained | **0.315** | **0.349** | **-0.555** | 1.000 |  |  |
| Number of species lost | **0.515** | **0.323** | **0.817** | 0.011 | 1.000 |  |
| Species turnover rate | 0.006 | -0.212 | 0.215 | 0.030 | 0.242 | 1.000 |

Supplementary references

1. Lehikoinen, A., Rintala, J., Lammi, E. & Pöysä, H. Habitat-specific population trajectories in boreal waterbirds: alarming trends and bioindicators for wetlands. *Anim. Conserv*. **19,** 88–95 (2016).
2. Elmberg, J., Nummi, P., Pöysä, H. & Sjöberg, K. Factors affecting species number and density of dabbling duck guilds in North Europe. *Ecography* **16,** 251–260 (1993).
3. Solonen, T. Structure and dynamics of the Finnish avifauna. *Memoranda Soc. Fauna Flora Fennica* **70**, 1–22 (1994).
4. Pöysä, H. Resource utilization pattern and guild structure in a waterfowl community. *Oikos* **40**, 295–307 (1983).
5. Kear, J. Ducks, geese and swans (ed. Kear, J.). Oxford: Oxford University Press (2005).
6. Holopainen, S. *et al*. Habitat use in ducks breeding in boreal freshwater wetlands: a review. *Eur. J. Wildl. Res*. **61**, 339–363 (2015).
7. Koskimies, P. & Väisänen, R. A. *Monitoring Bird Populations. A Manual of Methods Applied in Finland*. (Zoological Museum, Finnish Museum of Natural History, 1991).
8. Koskimies, P. & Pöysä, H. Waterfowl censusing in environmental monitoring: a comparison between point and round counts. *Ann. Zool. Fennici* **26**, 201–206 (1989).
9. Pöysä, H. *et al*. Habitat associations and habitat change: seeking explanation for population decline in breeding Eurasian wigeon Anas penelope. *Hydrobiologia* **785,** 207–217, doi: 10.1007/s10750-016-2922-4 (2017).
10. Suhonen, S. *et al*. Long term stability of boreal lake habitats and use by breeding ducks. *Boreal Environ. Res*. **16**(Suppl B), 71–80 (2011).
